# From Childhood Ingestion to Adult Obstruction: A Long-Retained Synthetic Gastric Foreign Body

**DOI:** 10.7759/cureus.113334

**Published:** 2026-07-24

**Authors:** Amar Habibovic, Naid Dizdarevic

**Affiliations:** 1 Department of Gastroenterology and Hepatology, University Clinical Center Tuzla, Tuzla, BIH

**Keywords:** endoscopic retrieval, enterotomy, gastric dysplasia, plastobezoar, polyurethane foam, s: gastric foreign body, small bowel obstruction

## Abstract

Foreign body ingestion occurs predominantly in children, and most objects pass spontaneously; only a minority require endoscopic or surgical intervention. Prolonged gastric retention of synthetic material with presentation in adulthood is exceptionally uncommon. A 26-year-old man, who recalled accidental ingestion of expanding construction foam at four to five years of age, was first evaluated by gastroenterology on initial presentation for long-standing epigastric pain, nocturnal discomfort, heartburn, acid regurgitation, reduced appetite, and intermittent vomiting. He had no significant recent weight loss and had always been slender (body mass index: 19 kg/m²). Esophagogastroduodenoscopy revealed a 2-cm angular ulcer, two smaller corpus ulcers, and a large green-black intragastric mass whose food-like appearance led it to be initially interpreted as undigested retained food. Gastric biopsies demonstrated chronic inflammatory and fibrotic changes, intestinal metaplasia, and moderate-to-severe epithelial dysplasia without invasive malignancy. The approximately 4-cm hard, presumed polyurethane-based plastobezoar resisted four endoscopic extraction procedures because it could neither be safely withdrawn through the gastroesophageal junction nor adequately fragmented with available devices. After the final attempt, it migrated into the ileum and produced acute mechanical small bowel obstruction. Open exploration identified the foreign body approximately 70 cm proximal to the ileocecal valve. It was removed through enterotomy without bowel resection because the intestine was viable. The operation lasted 50 minutes; oral intake resumed on postoperative day two, and the patient was discharged on postoperative day seven. Follow-up endoscopy approximately five months later showed only a small angular scar. Surveillance biopsies demonstrated chronic inactive gastritis with focal glandular atrophy and resolution of the previously observed dysplasia, with no ulceration or residual dysplasia. This case highlights the diagnostic difficulty of radiolucent synthetic material, the consequences of prolonged retention, and the need for timely transition to surgery when a large, noncompressible object cannot be safely removed endoscopically.

## Introduction

Foreign body ingestion is encountered most frequently in children, with the peak incidence between six months and six years of age. In adults, non-food foreign body ingestion is less common and is associated particularly with psychiatric illness, developmental impairment, alcohol intoxication, incarceration, edentulism, or accidental occupational exposure. Nevertheless, it may also occur accidentally in otherwise healthy individuals [1-4]. Across age groups, approximately 80%-90% of ingested foreign bodies pass through the gastrointestinal tract spontaneously, 10%-20% require endoscopic intervention, and fewer than 1% require surgery [2-5]. These figures describe the overall clinical course of ingestion; the true incidence or prevalence of foreign bodies retained in the stomach for years is unknown because such cases are limited mainly to isolated reports.

Coins, batteries, magnets, bones, dental prostheses, food boluses, and sharp metallic objects are among the commonly encountered items, while bezoars comprise conglomerates of indigestible organic or synthetic material. Most objects that traverse the esophagus pass uneventfully, but areas of physiological narrowing or angulation - including the pylorus, duodenal sweep, ileocecal region, and sites of pre-existing stenosis or prior surgery - are potential sites of impaction. Complications may occur early or after delayed retention and include pressure necrosis, erosions, ulceration, bleeding, obstruction, perforation, fistula formation, peritonitis, and aspiration [1-6].

The American Society for Gastrointestinal Endoscopy (ASGE) and European Society of Gastrointestinal Endoscopy (ESGE) recommend that management be guided by the object's size, shape, composition, location, time since ingestion, symptoms, and evidence of complications. Endoscopic retrieval is recommended for objects that are retained, symptomatic, hazardous, or unlikely to pass safely; surgical referral is indicated after failed endoscopic management or when intestinal obstruction, perforation, or another major complication develops [2,3]. Polyurethane foam and other construction materials are rarely reported and can form hard, irregular plastobezoars that are difficult to grasp or fragment [7-10]. Previously published cases have generally involved recent ingestion, followed by early obstruction, difficult endoscopic fragmentation, or perforation [7-10]. The present report differs by describing presumed retention from early childhood into adulthood, chronic ulcer-associated dysplasia that resolved on surveillance biopsy, and acute ileal obstruction after repeated retrieval attempts. Its educational message is that remote ingestion should remain in the differential diagnosis of otherwise unexplained chronic upper gastrointestinal symptoms and that failure of a planned endoscopic strategy should prompt timely reassessment and surgical consultation before uncontrolled fragmentation or migration occurs.

## Case presentation

A 26-year-old man first presented to the gastroenterology service at the initial gastroenterology evaluationbecause of long-standing epigastric abdominal pain, most pronounced at night, heartburn, acid regurgitation, reduced appetite, and intermittent vomiting. He recalled accidentally ingesting expanding polyurethane construction foam at approximately four to five years of age. The symptoms had reportedly occurred intermittently since childhood, although their exact onset and continuity could not be verified because contemporaneous pediatric records were unavailable. He lost some weight, but he denied significant recent weight loss and stated that he had always been slender from childhood. His body mass index was 19 kg/m². Childhood growth and psychomotor development were reported as normal. He denied pica, recurrent intentional foreign body ingestion, developmental disability, and psychiatric illness; no psychiatric diagnosis was documented in the available medical record.

Physical examination was unremarkable apart from moderate epigastric tenderness. Routine laboratory tests and abdominal ultrasonography showed no clinically significant abnormalities. Testing for *Helicobacter pylori* was negative. Pantoprazole 40 mg once daily was started.

Endoscopic and radiological evaluation

**Figure 1:**
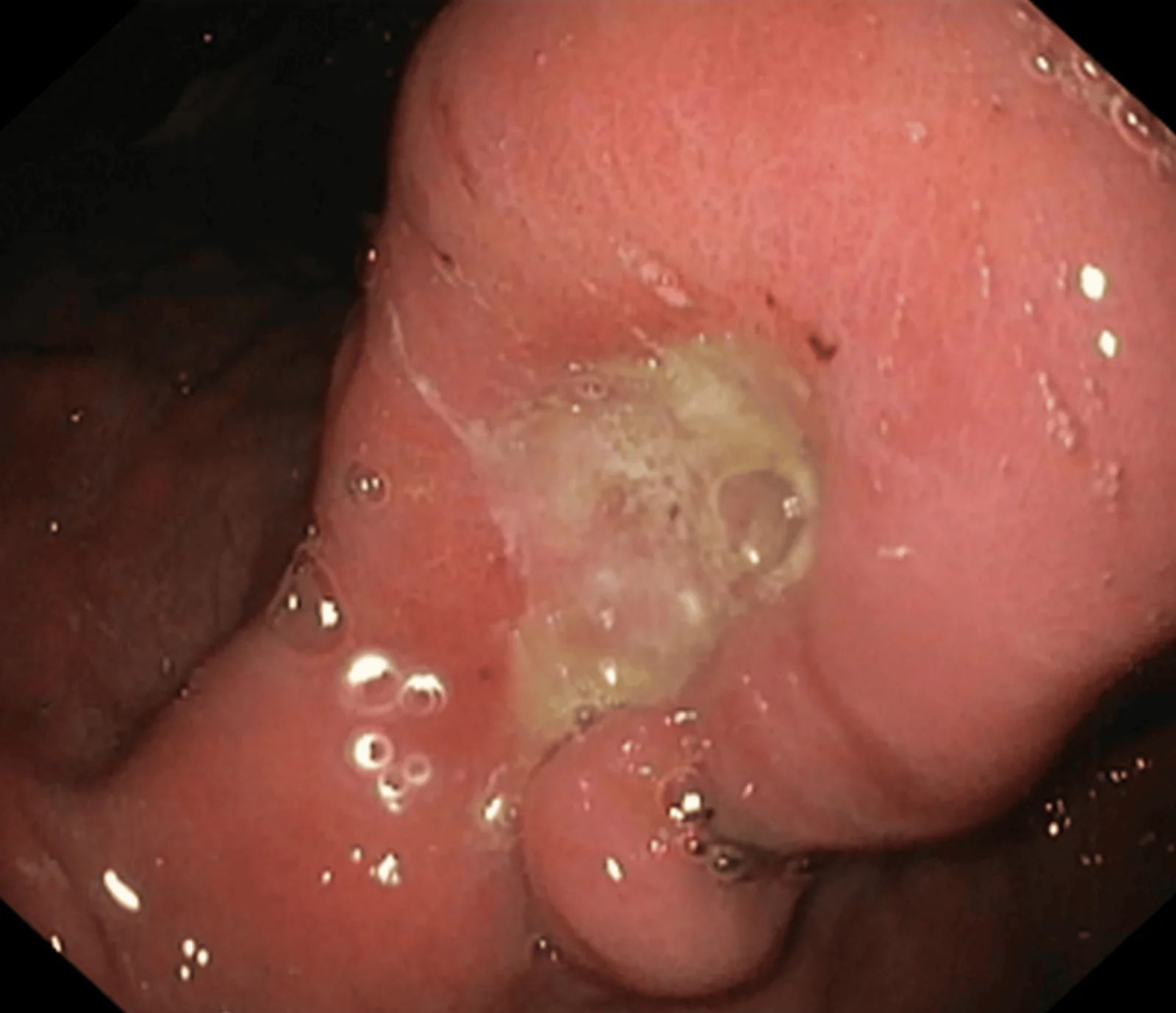
Initial esophagogastroduodenoscopy (EGD) showing the approximately 2-cm fibrin-covered ulcer at the gastric angulus

**Figure 2:**
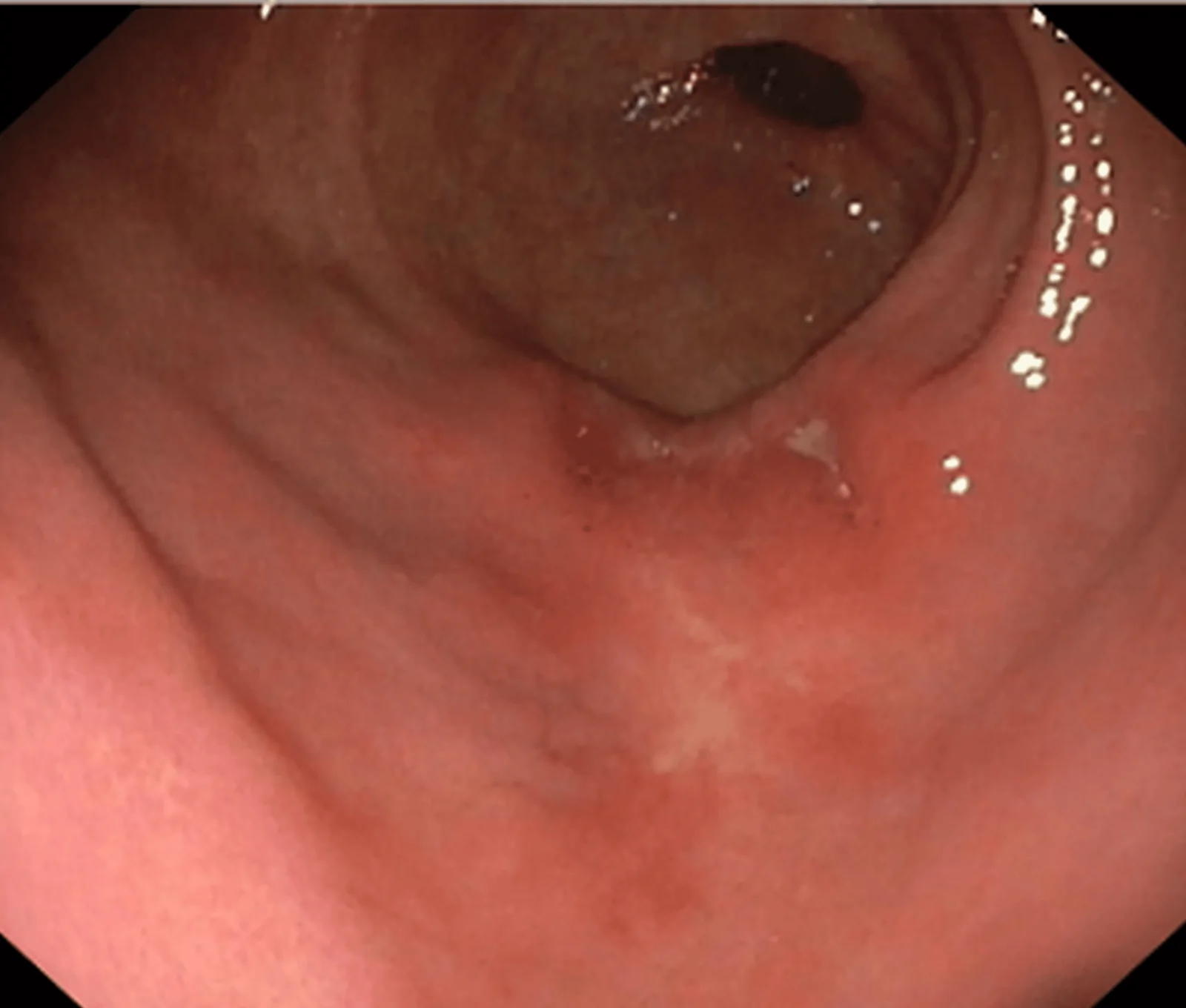
Endoscopic view showing two shallow stellate ulcers in the gastric corpus, each measuring approximately 1 cm, with fibrin-covered bases and surrounding mucosal erythema

**Figure 3:**
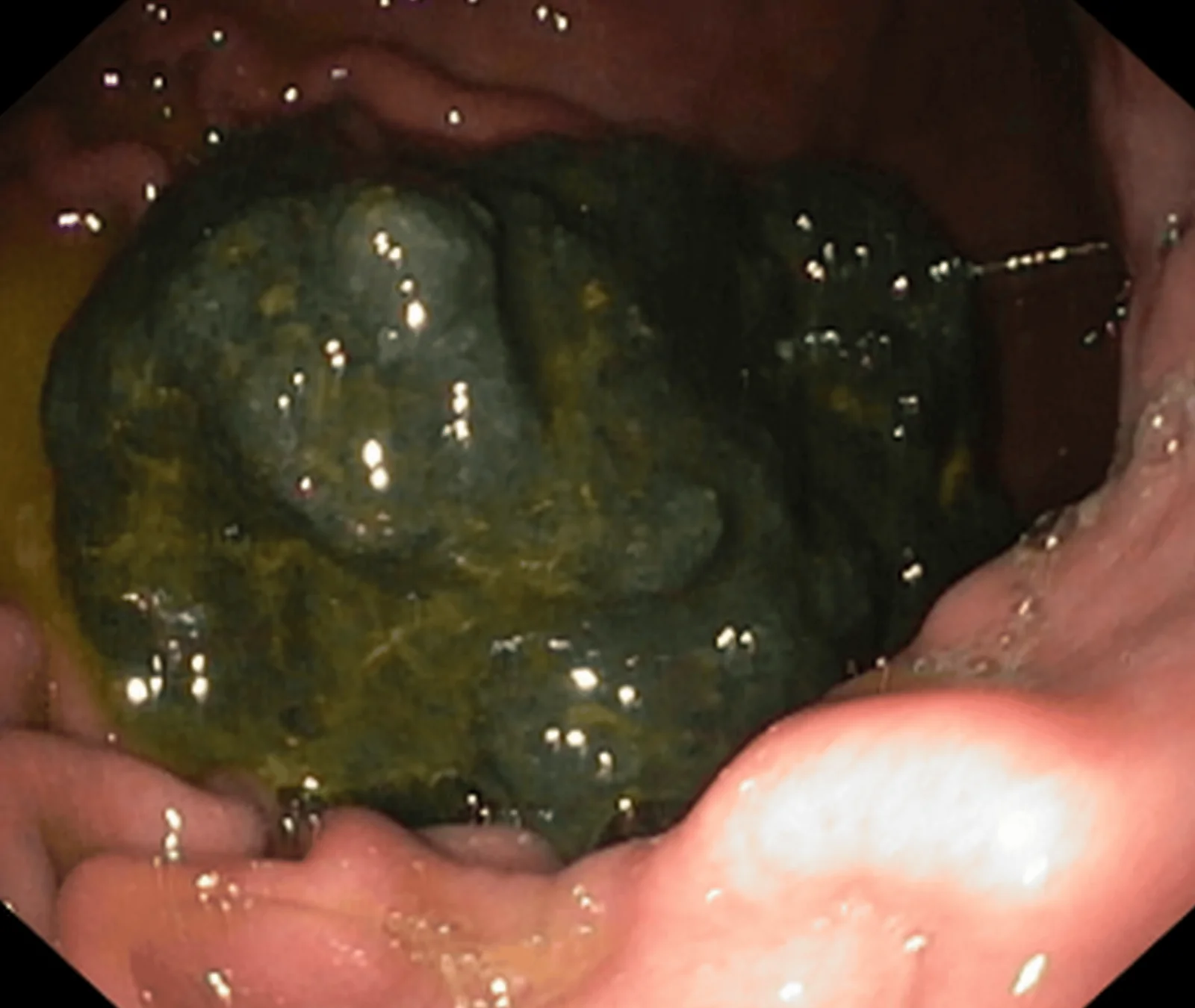
Endoscopic view demonstrating a large, irregular green-black presumed synthetic plastobezoar occupying a substantial portion of the gastric lumen Its food-like appearance initially led to its misinterpretation as retained undigested food.

**Figure 4:**
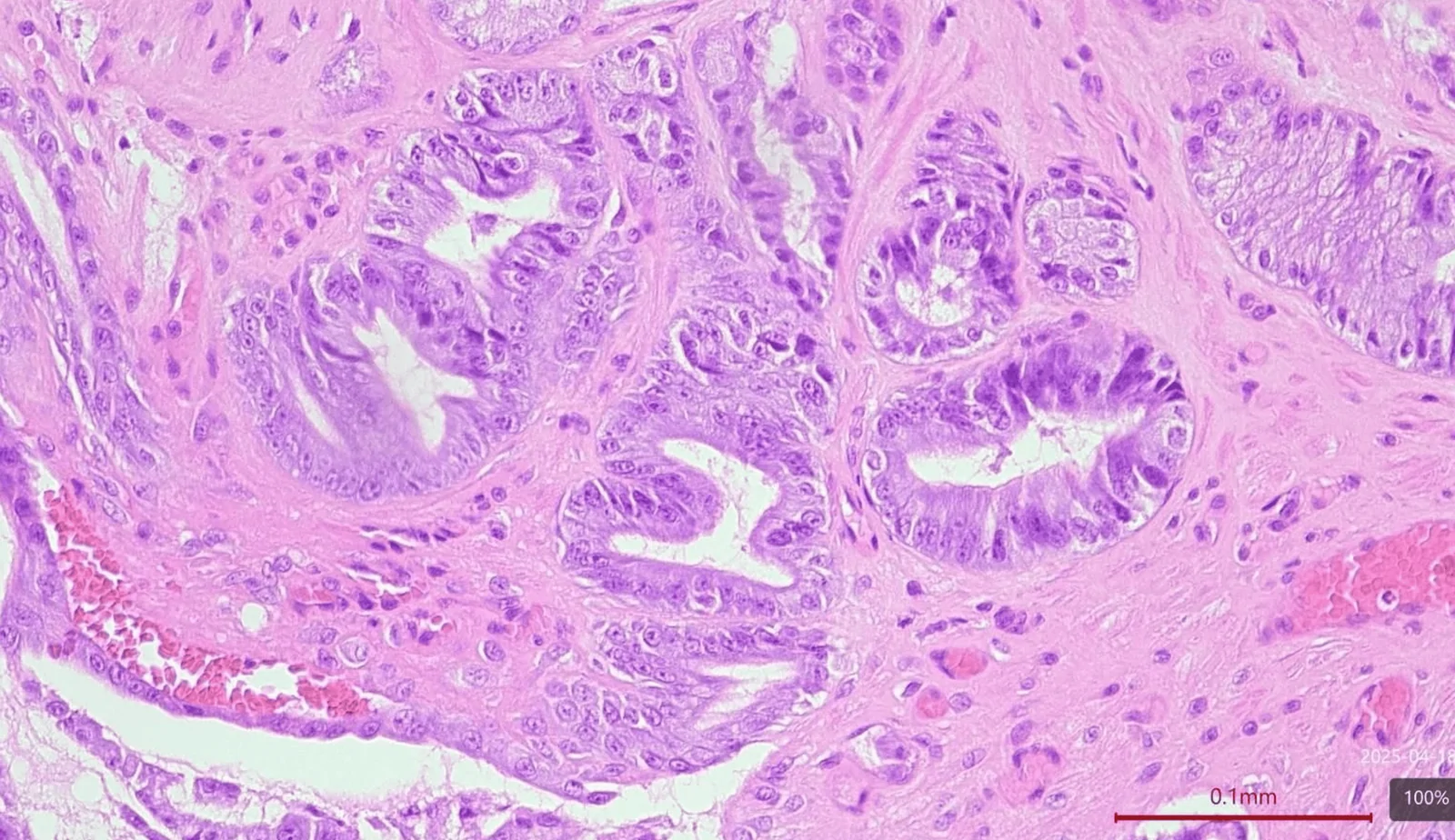
Gastric biopsy from the ulcer margin showing epithelial dysplasia in a background of chronic inflammatory and fibrotic change No invasive malignancy was identified.

Approximately two months after the initial gastroenterology evaluation, the first EGD showed a 2-cm oval ulcer at the gastric angulus with raised margins, surrounding erythema, and a fibrin-covered base (Figure 1). Two additional shallower stellate ulcers, each up to 1 cm, were present in the corpus (Figure 2). The gastric mucosa was diffusely hyperemic. An undigested-appearing intragastric structure was initially interpreted as possible retained food (Figure 3). Biopsies were obtained from the ulcerated mucosa. Histopathology demonstrated chronic inflammation and fibrosis with intestinal metaplasia and moderate-to-severe epithelial dysplasia, without invasive malignancy (Figure 4).

Six weeks after the initial EGD, repeat EGD again demonstrated the green-black intragastric mass, which had been visible at the initial examination, together with a persistent linear fibrin-covered angular ulcer and several small antral and corpus erosions. The object was considered the probable mechanical contributor to the mucosal injury. A snare removed approximately one-fifth of the mass, but the remaining object could not be passed through the cardia. An attempt to divide it mechanically was abandoned after the snare broke.

Twelve days after the second EGD, a third examination confirmed persistence of the hard oval gastric mass. Multiple snare extraction attempts were unsuccessful, although several small fragments were removed. Contrast-enhanced abdominal CT subsequently demonstrated thickened gastric folds and a fixed, bizarre mixed-density antral structure measuring approximately 42 × 27 mm, supporting the diagnosis of a foreign body.

Repeated endoscopic management was initially pursued because the object remained confined to the stomach, there was no evidence of perforation or intestinal obstruction, and complete endoscopic retrieval would have avoided operative morbidity in a young patient. Each attempt was intended either to reduce the mass to safely retrievable pieces or to remove it intact. However, the object's rigidity, smooth/irregular surface, inability to traverse the cardia, incomplete fragmentation, and eventual device failure progressively reduced the likelihood of safe endoscopic success. These cumulative technical limitations prompted the recommendation for surgical removal rather than further elective endoscopic manipulation.

Approximately seven weeks after the third EGD, during a fourth endoscopic retrieval attempt, the plastobezoar measured approximately 4 cm. Repeated extraction was again attempted, but the object became impacted at approximately 35 cm from the incisors and could not be safely withdrawn. Several additional fragments were detached. Subsequent inspection showed no visible esophageal mucosal injury. Nifedipine and nitroglycerin were used at the treating team's discretion during the overall retrieval strategy in an attempt to facilitate passage through the gastroesophageal junction; this was not a guideline-based standard maneuver and did not permit successful extraction. Surgical removal was recommended.

Acute obstruction and operative treatment

**Figure 5:**
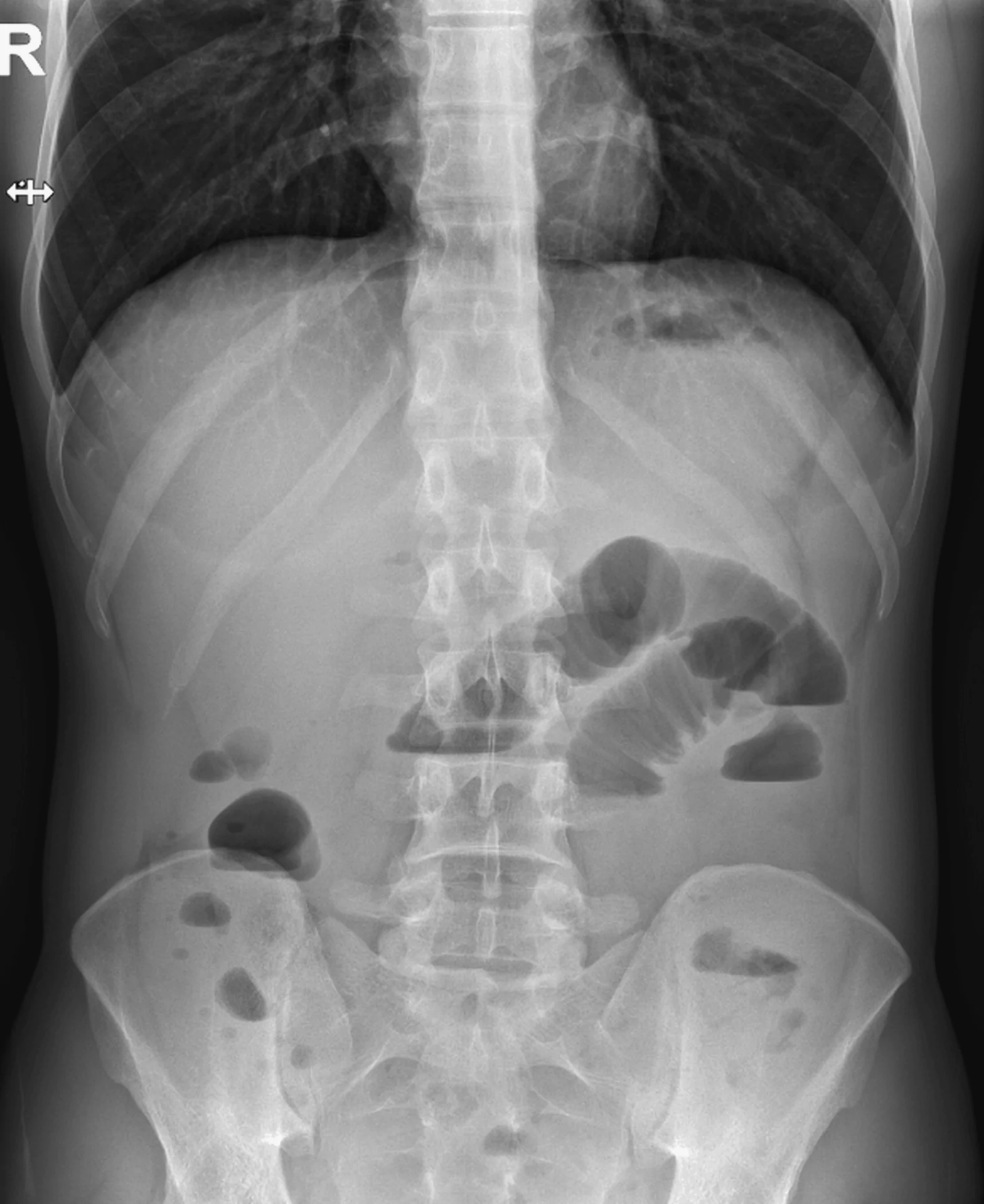
Upright plain abdominal radiograph showing multiple air-fluid levels and dilated small bowel loops measuring up to approximately 40 mm, consistent with mechanical small bowel obstruction

The day after the final endoscopic attempt, one day after the final endoscopic attempt, the patient developed abdominal pain, followed by repeated vomiting. He reported approximately 10 episodes of brown-yellow emesis. Plain abdominal radiography showed multiple air-fluid levels and small bowel dilatation up to approximately 40 mm (Figure 5). CT identified a 3 × 4 cm irregular, rounded structure containing small air foci, morphologically similar to the previously visualized gastric object, now located in a small bowel loop in the right pelvis. Proximal fluid- and gas-filled loops were dilated to approximately 4 cm, consistent with acute mechanical small bowel obstruction.

**Figure 6:**
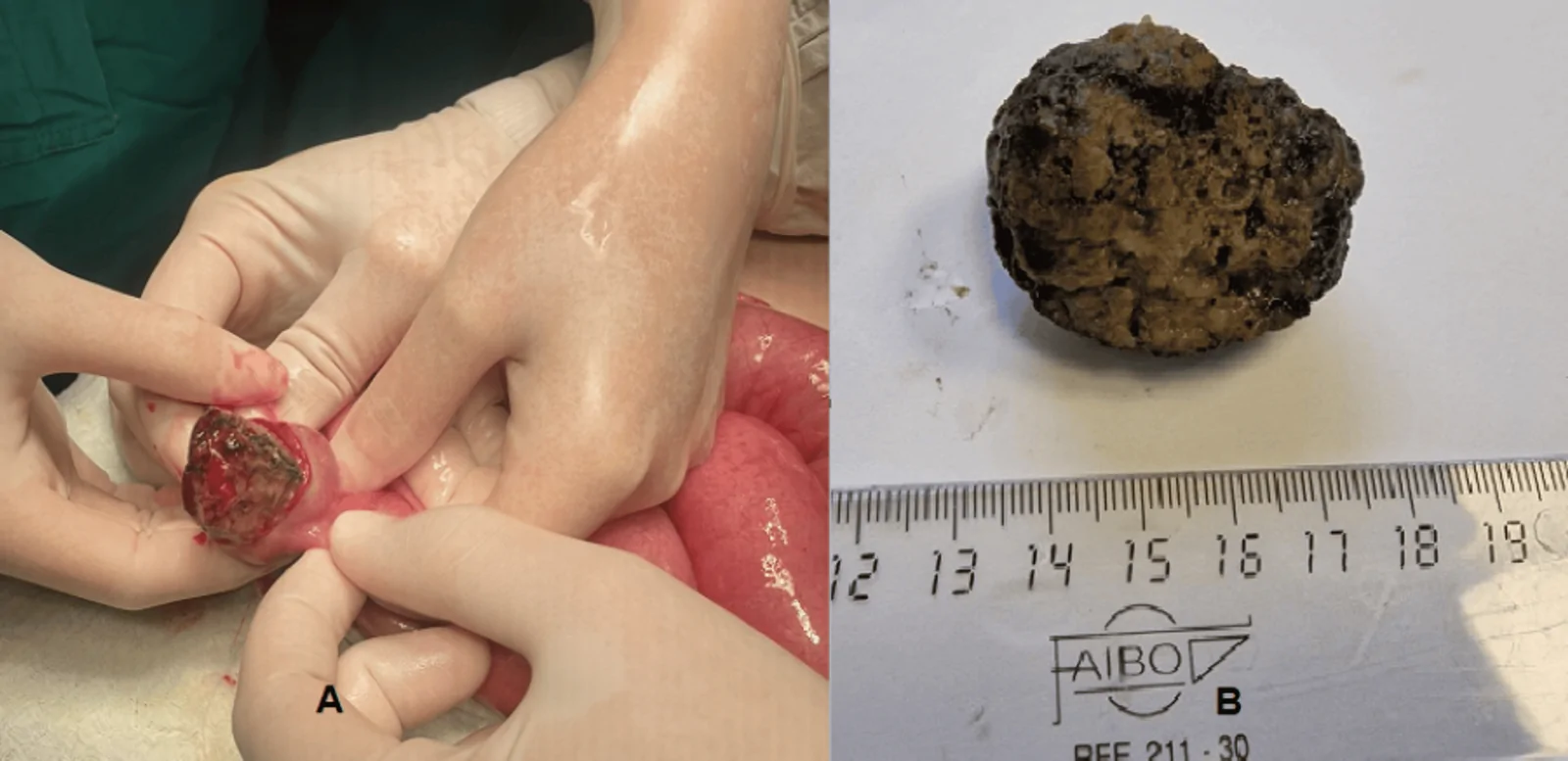
Operative removal and appearance of the synthetic foreign body (A) Intraoperative view showing extraction of the impacted synthetic foreign body through an ileal enterotomy approximately 70 cm proximal to the ileocecal valve. (B) The extracted walnut-sized synthetic foreign body (plastobezoar).

Emergency surgery was performed the following day. The operative time was 50 minutes. Following preparation of the operative field, a partial upper and lower midline laparotomy was performed. Exploration identified the foreign body in the ileum approximately 70 cm proximal to the ileocecal valve. Proximal small bowel loops were dilated, and the bowel wall was edematous, but the intestine remained viable, with no perforation, ischemia, or gangrene. An enterotomy was performed distal to the lower margin of the obstruction, and the walnut-sized synthetic foreign body was extracted through the enterotomy (Figures 6A-6B). The enterotomy was closed in two layers with interrupted 3-0 polyglactin sutures. Patency and integrity of the closure were confirmed. A small volume of ascitic fluid in the rectovesical pouch was evacuated, the abdominal cavity was irrigated, and a drain was placed in the rectovesical pouch. Hemostasis was satisfactory, the abdominal wall was closed anatomically, and the skin was closed with staples.

Histopathological examination of the extracted material was most consistent with inorganic material of synthetic origin and showed no neoplastic tissue. Dedicated chemical or spectroscopic materials analysis was not available; therefore, the exact polymer composition could not be independently confirmed.

Postoperative course and surveillance

**Figure 7:**
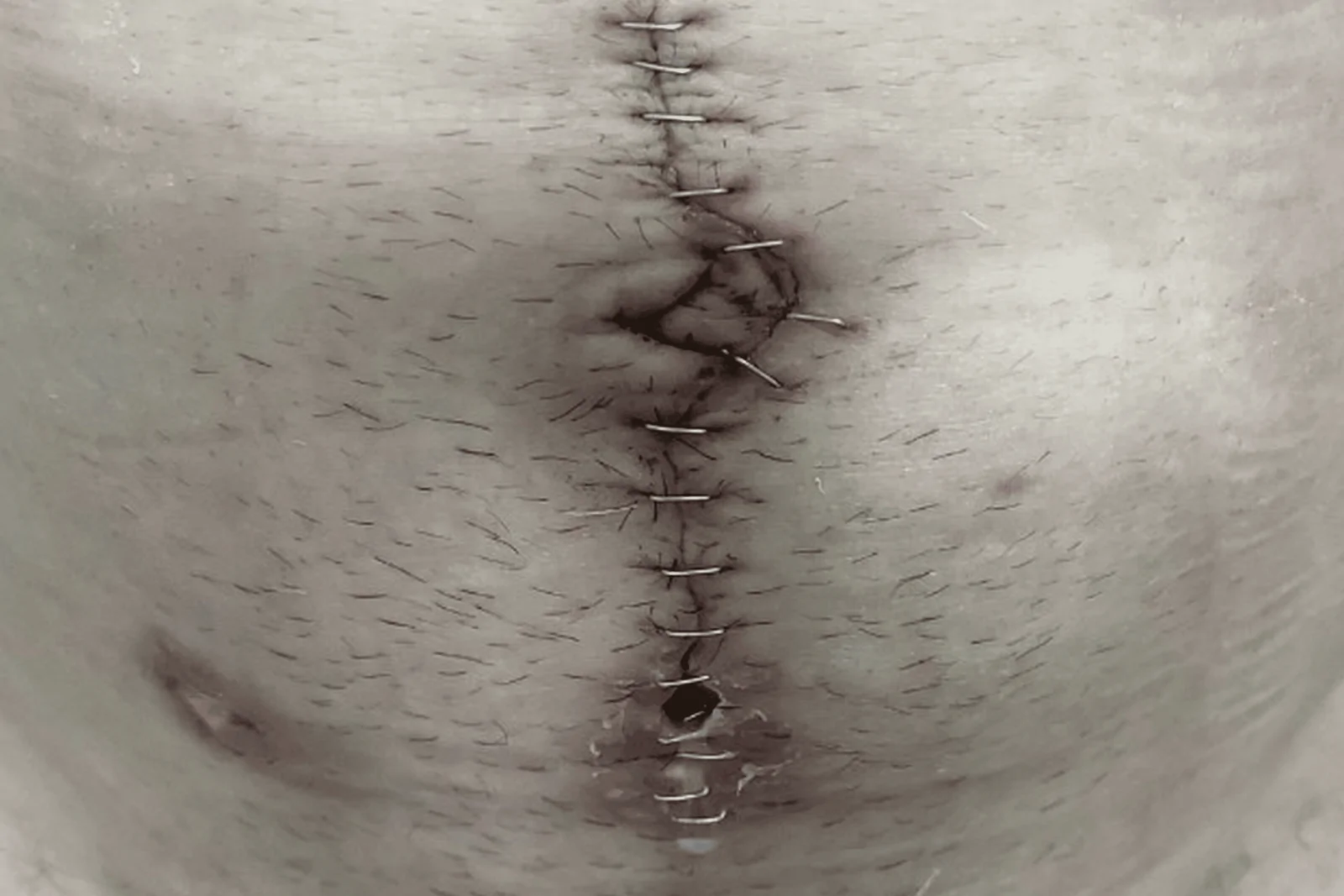
Postoperative midline incision showing mild localized erythema at the incision site and around several skin staples, with scant localized drainage Wound swab cultures were sterile, with no evidence of systemic infection or deep surgical site infection.

Recovery was favorable. Oral intake was resumed on postoperative day two. The patient developed mild localized erythema at the incision site and around several skin staples, accompanied by scant localized drainage; wound swab cultures were sterile, and there was no evidence of systemic infection or deep surgical-site complication (Figure 7). He was discharged after a seven-day hospital stay.

**Figure 8:**
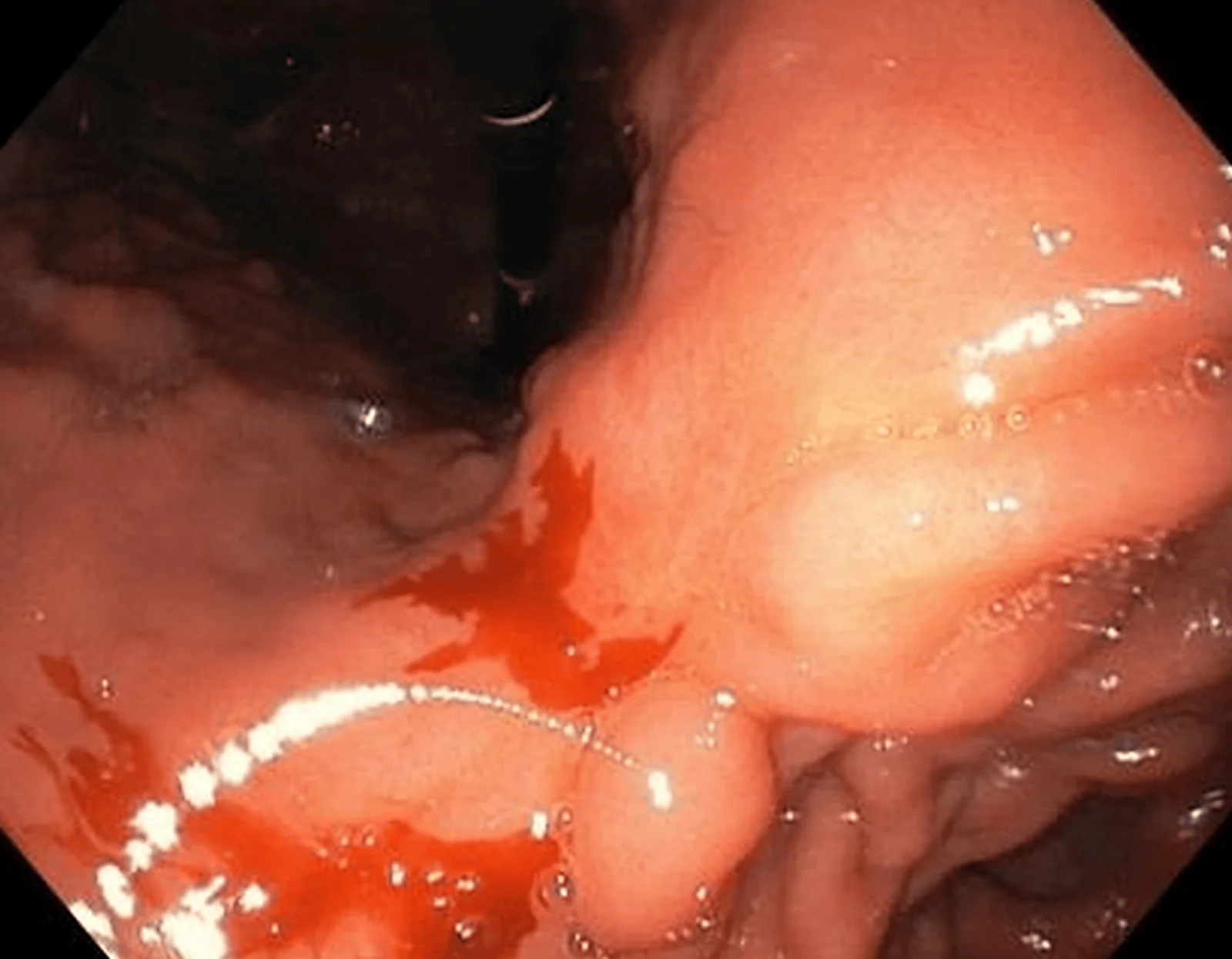
Follow-up EGD showing a small residual scar at the site of the previous angular ulcer before targeted surveillance biopsies

Surveillance EGD was performed approximately five months after surgery. The esophagus was normal. The stomach was insufflated normally and contained no retained material. At the previous angular ulcer site, only a small scar remained, and targeted biopsies were obtained because the preoperative specimens had shown moderate-to-severe dysplasia (Figure 8). A small sliding hiatal hernia was noted. The pylorus, duodenal bulb, and second portion of the duodenum were normal.

Microscopic examination of the surveillance biopsies showed chronic inflammatory changes without active inflammation, with glandular atrophy in one specimen. No ulceration or dysplasia was identified. The final diagnosis was chronic gastritis. The patient reported clinical improvement without recurrent obstructive symptoms. Written informed consent was obtained for publication of the clinical information and images. Table 1 presents the clinical timeline of diagnostic evaluation, endoscopic retrieval attempts, surgical treatment, and follow-up.

**Table 1:** Clinical timeline of diagnostic evaluation, endoscopic retrieval attempts, surgical treatment, and follow-up

Date/period	Clinical event
Early childhood	Patient-reported accidental ingestion of expanding construction foam; contemporaneous medical documentation was unavailable.
Initial presentation	First gastroenterology evaluation for long-standing epigastric and reflux symptoms, reduced appetite, and intermittent vomiting; no significant recent weight loss; BMI 19 kg/m².
Approximately two months later	First EGD: 2-cm angular ulcer, two smaller corpus ulcers, and a food-like intragastric structure; biopsies showed intestinal metaplasia and moderate-to-severe epithelial dysplasia without invasive malignancy.
Six weeks after the first EGD	Second EGD: persistent ulcer and approximately 4-cm mass; about one-fifth was removed, but the remaining object could not pass through the cardia; the snare broke during attempted fragmentation.
Twelve days after the second EGD	Third EGD: repeated snare retrieval was unsuccessful; several small fragments were removed. CT subsequently confirmed a fixed mixed-density gastric foreign body.
Approximately seven weeks after the third EGD	Fourth EGD: the plastobezoar again could not be extracted and became impacted at 35 cm from the incisors; additional fragments were removed without visible esophageal injury.
The following day	Acute abdominal pain and approximately 10 episodes of vomiting. Radiography and CT demonstrated distal migration of the object with mechanical small bowel obstruction.
One day after the onset of obstructive symptoms	A 50-minute partial upper and lower midline laparotomy identified the object in the ileum, approximately 70 cm proximal to the ileocecal valve. Enterotomy and extraction were performed without bowel resection.
Postoperative course	Oral intake resumed on postoperative day two. Mild localized incisional erythema and scant drainage occurred; wound swabs were sterile. The patient was discharged on postoperative day seven.
Approximately five months after surgery	Follow-up EGD showed a healed angular ulcer with a small scar. Targeted biopsies demonstrated chronic gastritis with focal glandular atrophy and no ulceration or residual dysplasia.

## Discussion

This case combines several unusual features: presumed retention of synthetic material from early childhood into adulthood, chronic ulcer-associated mucosal injury, repeated technical failure of endoscopic extraction, migration after manipulation, and acute ileal obstruction. Foreign body ingestion is overwhelmingly more common in early childhood, whereas delayed adult presentation of an object reportedly swallowed at four to five years of age is exceptional. Because long-retained synthetic gastric foreign bodies are described principally in individual reports, a reliable population incidence or prevalence cannot be stated. The broader and better-supported epidemiological estimates - spontaneous passage in 80%-90%, endoscopic intervention in 10%-20%, and surgery in fewer than 1% - should not be misrepresented as the prevalence of long-term retention [2-5].

Once an object has passed the esophagus, the pylorus, duodenal sweep, ileocecal region, and pathological narrowings are recognized sites where progression may fail. Complications depend on size, shape, chemical properties, and duration of contact. Acute risks include impaction, aspiration, bleeding, perforation, and obstruction; prolonged pressure or friction may produce erosions, ulcers, necrosis, fistulae, or chronic inflammatory and proliferative mucosal changes [1-6]. In the present case, the object remained gastric long enough to be associated with multiple ulcers and then obstructed the distal ileum after migration. Table 2 presents the comparison with previously reported polyurethane and plastic bezoars.

**Table 2:** Comparison of previously reported polyurethane and plastic bezoars

Report	Patient/exposure	Presentation/complication	Management	Relevance
Medani et al., 2009 [7]	44-year-old man; recent polyurethane foam ingestion	Fragment migrated and caused small bowel obstruction	Laparotomy and surgical retrieval	Demonstrates obstruction after distal migration of polyurethane material.
Girardin et al., 2011 [8]	61-year-old man; symptoms three weeks after ingesting self-expanding spray foam	Approximately 8-cm hard gastric bezoar with three deep corpus ulcers	Two prolonged EGDs; mechanical gutter creation and fragmentation; complete endoscopic extraction	Authors avoided leaving fragments <3 cm because of small bowel ileus risk.
Misra et al., 2006 [9]	11-year-old child; ingested plastic threads	Large plastobezoar occupying stomach; chronic antral irritation, ulceration, and hyperplastic polyps	Endoscopic management described	Supports chronic mucosal injury from retained plastic material.
Ziaja et al., 2020 [10]	39-year-old man; polyurethane system ingestion	Hard esophagogastric bezoar, obstruction, gastric microperforations, and peritonitis	Emergency open surgery	Shows severe pressure-related and perforating complications and difficulty of endoscopic grasping.
Present case	26-year-old man; presumed ingestion at age 4-5 years	Long gastric retention, ulcers with dysplasia, failed extraction, migration, and ileal obstruction	Four EGDs followed by 50-minute midline laparotomy and ileal enterotomy	Exceptionally delayed adult presentation; surveillance biopsy showed healing and no residual dysplasia.

Endoscopic management

American Society for Gastrointestinal Endoscopy (ASGE) and European Society of Gastrointestinal Endoscopy (ESGE) guidance supports endoscopic removal of retained upper gastrointestinal foreign bodies when size, shape, composition, symptoms, or complication risk make spontaneous passage unsafe. Selection of snares, baskets, nets, forceps, caps, overtubes, or other protective devices should be individualized [2,3]. Medium-sized blunt gastric objects may be removed non-urgently, whereas sharp objects, batteries, magnets, and large or long objects generally require more urgent intervention. Surgery is appropriate after failed endoscopic removal or when obstruction or perforation occurs.

Polyurethane plastobezoars present special mechanical challenges. Expansion followed by hardening may produce a large, aerated but rigid object with a smooth or irregular surface, limiting snare purchase and making controlled fragmentation difficult. Girardin et al. required two procedures and approximately seven hours of total endoscopy time to remove an 8-cm foam bezoar; importantly, they avoided leaving fragments smaller than 3 cm because these could migrate and produce ileus [8]. In our case, multiple devices failed, one snare broke, and the object repeatedly could not traverse the gastroesophageal junction. The subsequent ileal obstruction illustrates precisely this migration hazard. Although manipulation may have contributed, spontaneous migration cannot be excluded, and causality cannot be established from temporal association alone.

The unsuccessful use of nifedipine and nitroglycerin in this case should be interpreted cautiously. These agents are not recommended by ASGE or ESGE as standard adjuncts for extracting a large gastric foreign body through the cardia. Their use reflected an individualized attempt by the treating team and should not delay definitive endoscopic or surgical management. After repeated inability to achieve safe extraction, earlier operative consultation would have limited further manipulation and the risk of distal migration.

Open, laparoscopic, and laparoscopic-assisted surgery

No current ASGE or ESGE guideline designates either a supraumbilical-to-pubic mini-laparotomy or exploratory laparoscopy with exteriorization through a suprapubic Pfannenstiel incision as the universal gold-standard operation for an obstructing intestinal foreign body. The endoscopy guidelines define when surgical referral is required but do not prescribe a specific abdominal incision [2,3]. The operative route should instead be individualized according to hemodynamic stability, degree of bowel distension, certainty of localization, suspected ischemia or perforation, prior surgery, available expertise, and the anticipated need for resection.

Laparoscopy may permit full exploration, localization, and minimally invasive removal in selected stable patients, with less postoperative pain, earlier recovery, fewer wound complications, and a shorter hospital stay. A laparoscopic-assisted technique can identify the involved loop and exteriorize it through a small protected suprapubic or periumbilical incision, allowing controlled enterotomy and closure outside the abdomen. This can combine diagnostic visualization with limited access trauma. However, markedly distended and edematous bowel reduces working space and increases the risk of iatrogenic enterotomy, missed injury, or conversion. Evidence from small bowel obstruction literature supports careful patient selection rather than a single mandated approach; even in better-studied adhesive obstruction, World Society of Emergency Surgery (WSES) guidance warns that laparoscopy in the presence of markedly distended bowel may increase bowel injury risk [11].

Open midline laparotomy offers rapid, broad exposure, allows the entire small bowel to be examined manually, and facilitates immediate enterotomy or resection when viability is uncertain. Its disadvantages include greater incision-related pain, wound morbidity, recovery time, and subsequent adhesion formation. In this patient, acute mechanical obstruction, dilated edematous loops, uncertainty regarding the exact intraoperative condition of the bowel, and the need for prompt definitive removal supported the chosen partial upper and lower midline laparotomy. Because the bowel was viable and intact, enterotomy with two-layer closure was sufficient, and resection was avoided. The favorable 50-minute operation, resumption of oral intake on day two, and discharge on day seven support the safety of this individualized choice, although they do not establish superiority over a laparoscopic-assisted approach.

Gastric dysplasia and follow-up

Preoperative ulcer biopsies showed intestinal metaplasia and moderate-to-severe epithelial dysplasia in a background of chronic inflammation and fibrosis. These findings required follow-up because sampling error can occur in ulcerated lesions and because dysplasia is clinically relevant even when invasive malignancy is not identified. The foreign body plausibly contributed to repeated pressure and friction at the angular mucosa, but a single case cannot demonstrate that it caused the dysplasia. Rare descriptions of gastric neoplasia adjacent to chronic foreign bodies likewise establish association rather than causation [12].

Targeted repeat biopsies were therefore obtained approximately five months after surgery. They showed chronic inactive gastritis, with glandular atrophy in one specimen, but no residual ulceration or dysplasia. This supports mucosal healing after removal and provides reassuring short-term histological surveillance. Given the original dysplasia and intestinal metaplasia, continued follow-up should be individualized according to the completeness and quality of baseline sampling, local gastric premalignant lesion protocols, family history, and any recurrent endoscopic abnormality.

Limitations

This report has several limitations. First, childhood ingestion was based on patient recall, and no contemporaneous pediatric documentation or prior imaging was available; the precise retention duration therefore cannot be proven. Second, the exact timing and mechanism of migration from the stomach into the small bowel cannot be determined, and endoscopic manipulation can only be considered a possible contributor. Third, the extracted object underwent histopathological assessment but not dedicated chemical or spectroscopic analysis, so the polyurethane composition remains presumed from the history and macroscopic characteristics. Fourth, this is a single case and cannot establish causation between chronic foreign-body contact and epithelial dysplasia or determine the optimal operative approach. Finally, follow-up is limited to one clinical and endoscopic assessment approximately five months after surgery; longer surveillance would strengthen conclusions about recurrence and mucosal outcome.

## Conclusions

A synthetic gastric foreign body may remain unrecognized for years, produce nonspecific upper gastrointestinal symptoms and chronic ulcerative mucosal injury, resist endoscopic retrieval, and cause acute small bowel obstruction after distal migration. Remote foreign body ingestion should therefore be considered in patients with longstanding unexplained upper gastrointestinal symptoms, even when presentation occurs in adulthood. A radiolucent or mixed-density intragastric structure should not automatically be labeled as retained food when it persists across examinations. Endoscopic strategy must account for object size, rigidity, surface characteristics, and the ability to prevent uncontrolled fragment migration. When safe extraction cannot be achieved after a well-planned attempt, early surgical consultation is preferable to repeated manipulation. Neither open nor laparoscopic surgery is universally mandated; the approach should be individualized according to bowel distension, localization, suspected complications, patient stability, and local expertise. The mucosal injury and dysplasia in this single patient represent an observed association rather than proof of causation. Nevertheless, dysplasia detected adjacent to a chronically retained foreign body requires follow-up endoscopic and histological surveillance; in this patient, repeat biopsies after removal showed healing and no residual dysplasia.
